# Gene polymorphisms in association with emerging cardiovascular risk markers in adult women

**DOI:** 10.1186/1471-2350-11-6

**Published:** 2010-01-15

**Authors:** Amy Z Fan, Ajay Yesupriya, Man-huei Chang, Meaghan House, Jing Fang, Renée Ned, Donald Hayes, Nicole F Dowling, Ali H Mokdad

**Affiliations:** 1 National Center for Chronic Disease Prevention and Health Promotion, Centers for Disease Control and Prevention, 1600 Clifton Road, Atlanta, GA 30333, USA; 2 School of Public Health, Emory University, 1518 Clifton Road NE, Atlanta, GA 30329, USA; 3 Institute for Health Metrics and Evaluation, University of Washington, 2301 5th Avenue Suite600, Seattle, WA 98121, USA

## Abstract

**Background:**

Evidence on the associations of emerging cardiovascular disease risk factors/markers with genes may help identify intermediate pathways of disease susceptibility in the general population. This population-based study is aimed to determine the presence of associations between a wide array of genetic variants and emerging cardiovascular risk markers among adult US women.

**Methods:**

The current analysis was performed among the National Health and Nutrition Examination Survey (NHANES) III phase 2 samples of adult women aged 17 years and older (sample size n = 3409). Fourteen candidate genes within *ADRB2, ADRB3, CAT, CRP, F2, F5, FGB, ITGB3, MTHFR, NOS3, PON1, PPARG, TLR4*, and *TNF *were examined for associations with emerging cardiovascular risk markers such as serum C-reactive protein, homocysteine, uric acid, and plasma fibrinogen. Linear regression models were performed using SAS-callable SUDAAN 9.0. The covariates included age, race/ethnicity, education, menopausal status, female hormone use, aspirin use, and lifestyle factors.

**Results:**

In covariate-adjusted models, serum C-reactive protein concentrations were significantly (P value controlling for false-discovery rate ≤ 0.05) associated with polymorphisms in *CRP *(rs3093058, rs1205)*, MTHFR *(rs1801131)*, and ADRB3 *(rs4994). Serum homocysteine levels were significantly associated with *MTHFR *(rs1801133).

**Conclusion:**

The significant associations between certain gene variants with concentration variations in serum C-reactive protein and homocysteine among adult women need to be confirmed in further genetic association studies.

## Background

Coronary heart disease and stroke remain the leading causes of death and disability for men and women in the United States [1,2]. Atherosclerotic cardiovascular disease, which affects the heart, brain, and peripheral circulation, is responsible for the majority of the cases [3]. Traditional risk factors cannot fully account for the variation in the prevalence of heart disease in the general population. Some biomarkers, including C-reactive protein, fibrinogen, uric acid, and homocysteine, are among those which have been proposed as potential modifiable risk factors/markers in the last two decades.

Inflammation plays a key role in the initiation, progression, and outcome of atherosclerosis [4,5]. In prospective studies, markers of inflammation such as C-reactive protein (CRP) and fibrinogen have been found to be predictive of atherosclerosis and an increased risk of CVD events [4-16]. Elevated levels of plasma homocysteine and serum uric acid have been associated with increased risk of cardio- or cerebrovascular disease [17-21]. In addition, these emerging cardiovascular risk biomarkers influence each other and are correlated with conventional risk factors/markers such as high blood pressure or hyperlipidemia [22-24].

The concentrations of all four emerging biomarkers (CRP, fibrinogen, uric acid, homocysteine) are caused by complex interactions between environmental risk factors and predisposing genes. The candidate genes in this study, i.e., *ADRB2, ADRB3, CAT, CRP, F2, F5, FGB, ITGB3, MTHFR, NOS3, PON1, PPARG, TLR4*, and *TNF*, have been suggested to confer excess risk of cardiovascular disease, although the results are inconsistent from different association studies [25]. These candidate genes were selected from a set of variants that were previously genotyped in the NHANES III genetic data [26] and were identified from systematic literature reviews of previously published candidate gene association studies and meta-analyses [27-33].

The evidence on the associations of four novel risk factors/markers with these genes may help identify intermediate pathways of CVD susceptibility in the general population. For example, because genetic traits confer a risk of inflammation, common gene polymorphisms (> 1% frequency in the general population) may explain an individual's likelihood of developing inflammation or why some have a greater inflammatory response than others [34-36]. The National Health and Nutrition Examination Survey (NHANES) III DNA bank offers a unique sample to carry out this analysis as it has a large sample size and a diversity of ages, races and ethnicities that is representative of the US population. We examined the presence and magnitude of associations between candidate genetic variants (n = 27) within *ADBR2, ADBR3, CAT, CRP, F2, F5, FGB, ITGB3, MTHFR, NOS3, PON1, PPARG, TLR4*, and *TNF *[26,37] and four cardiovascular risk markers (CRP, fibrinogen, homocysteine, and uric acid) among adult women.

## Methods

### Study Sample

Participants took part in the second phase (1991-1994) of the Third National Health and Nutrition Examination Survey (NHANES III). The NHANES are complex, multistage cross-sectional sample surveys conducted by the National Center for Health Statistics (NCHS) of the Centers for Disease Control and Prevention (CDC). NHANES III included a stratified multistage probability design to provide national estimates of common diseases and their respective risk factors for the civilian non-institutionalized population in the United States ages two months or older, from 1988 through 1994. Data collection for NHANES occurs at three levels: a brief household screener interview, an in-depth household survey interview, and an extensive medical examination [38]. Population weights are calculated for each individual to make the data representative of the US population. In the second phase of NHANES III, white blood cells were frozen and cell lines were immortalized with the Epstein- Barr virus, creating a DNA bank. The current analysis was performed among adult women aged 17 years and older (n = 3409). The study was approved by the NCHS Ethics Review Board. NHANES III DNA bank, selection of candidate genes and variants, genotyping methods, and quality controls are detailed elsewhere [26].

### Genotyping Methods

Most genotypes were assayed either by TaqMan (5' nuclease assay; Applied Biosystems, Foster City, CA) or by the MGB Eclipse Assay (3' hybridization triggered fluorescence reaction; Nanogen, Bothwell, WA). *ADRB2 *and *F2 *were genotyped using pyrosequencing. Water controls and DNA samples with known genotypes, purchased from Coriell Cell Repository (Camden, NJ) were included on each well plate [26].

### Biochemical Analysis

The laboratory procedures for the assessment of serum C reactive protein, serum uric acid, serum homocysteine and plasma fibrinogen are available from the NCHS website [39].

### Covariates

Potential confounders of the gene-outcome relationship were selected a priori. Demographic characteristics include age (17-40 yrs, 41-59 yrs, 60 + yrs), race-ethnicity (non-Hispanic white, non-Hispanic black, Mexican American), and educational attainment (< 12 yrs, 12 yrs, college and above). Lifestyle factors include smoking status (current, former, never), drinking status (lifetime abstainer, former drinker, current drinker), total dietary fiber intake (≥ or < 7 gm/1000 kcal), total energy intake (≥ or < 1600 kcal per day), and physical activity (none, low, high). Other covariates include BMI (< 25 kg/m^2^, 25-29.9 kg/m^2^, ≥ 30 kg/m^2^), menopausal status (yes/no), female hormone use (yes/no), and aspirin use (yes/no). Details on descriptions of covariates are available elsewhere [32].

### Statistical Analysis

Weighted allele frequencies of genetic variants in the US population by race/ethnicity using the NHANES III phase 2 DNA bank have been presented elsewhere [26].

Deviations from Hardy-Weinberg proportions were tested in a standard unweighted analysis using Chi-square goodness-of-fit approach. Point estimates and 95% confidence intervals for the distribution of the demographic, lifestyle and biomarker variables were calculated. The Taylor series linearization approach was used to estimate the variance for standard errors.

Adjusted means of the outcome variables (inflammation markers) by gene variants were obtained from multiple linear regression models. Candidate covariates/potential confounders included age, race/ethnicity, education, menopausal status, female hormone use, smoking status, drinking status, dietary fiber intake, total energy intake, physical activity, body mass index, and aspirin use. However, only significant covariates "in the crude models" were retained in fully-adjusted models for a specific marker predicted by certain genetic variants. For CRP, total energy intake was excluded; for fibrinogen, dietary fiber intake was excluded; for homocysteine, drinking status was excluded; and for uric acid, drinking status and aspirin use were excluded. Minimally adjusted models were also presented with adjustment of only race/ethnicity. We presented adjusted means by genotype [40] and made groupwise comparisons. A P value ≤ 0.05 of the Satterthwaite-adjusted F-statistic in fully adjusted models was considered as statistically significant. False Discovery Rate (FDR)-adjusted P values (adjusted for a maximum of 27 tests) are presented along with unadjusted P values from Wald Chi-square tests. All outcome variables were right-skewed and were thus log-transformed before analysis. The analyses were performed in SAS-callable SUDAAN 9.01 (Research Triangle Institute, NC, 2007) to account for the complex sampling design, non-response, and sample weights for Genetic Component of NHANES III.

## Results

Characteristics of the study population based on the 3,409 participants are described in Table 1. The weighted frequency distribution was 81.3% non-Hispanic white, 13.2% non-Hispanic black, and 5.6% Mexican American. Current smokers accounted for 25.7% of the study population, while 43.3% were current drinkers. Approximately 41% of women have undergone menopause; and about 16% were currently using any form of female hormone. The correlation matrix for the four logarithm-transformed biomarkers is shown in Additional File 1: Table S1. The Pearson correlation coefficients ranged from 0.04 to 0.39.

**Table 1:** Characteristics of study population of US women, Third National Health and Nutrition Examination Survey, Phase 2 (1991-1994)

	Percent (Standard Error)
	N = 3,409
Age	
17-40	45.6 (1.8)
41-59	30.2 (1.6)
60+	24.2 (2.2)
Race/Ethnicity	
Non-Hispanic white	81.3 (1.8)
Non-Hispanic black	13.2 (1.7)
Mexican American	5.6 (0.8)
Educational attainment	
< 12 yrs	22.1 (1.6)
12 yrs	37.5 (1.4)
College or above	40.4 (2.4)
BMI (kg/m^2^)	
< 25	48.1 (1.9)
25-29.9	25.4 (0.8)
≥ 30	26.5 (1.4)
Smoking status	
Current smoker	25.7 (1.5)
Former smoker	18.7 (1.4)
Never smoker	55.6 (1.8)
Drinking status	
Lifetime abstainer	17.9 (1.7)
Former drinker	38.8 (1.3)
Current drinker	43.3 (2.2)
Total dietary fiber intake	
≥ 7 gm/1000 kcal	59.8 (1.9)
< 7 gm/1000 kcal	40.2 (1.9)
Total energy intake	
≥ 1600 kcal	56.4 (1.4)
< 1600 kcal	43.6 (1.4)
Physical activity	
None	22.7 (2.1)
Low	35.3 (1.1)
High	42.0 (1.9)
Menopausal status	
Yes	40.7 (2.2)
No	59.3 (2.2)
Aspirin use	
None	68.0 (1.2)
< 1 day/month	30.1 (1.1)
≥ 1 day/month	1.8 (0.4)
Female hormone use	
Yes	16.0 (1.4)
No	84.0 (1.4)
Serum C-reactive protein (mg/dL)^a^	0.33 (0.31,0.35)
Plasma fibrinogen (g/L)^a^	3.00 (2.95, 3.06)
Serum homocysteine (umol/L)^a^	8.17 (8.01, 8.33)
Serum uric acid (umol/L)^a^	270 (265, 276)

^a ^Data are geometric mean (95% confidence interval).

In fully-adjusted models, serum C-reactive protein concentrations were significantly associated with polymorphisms in *CRP *(rs3093058, rs1205)*, MTHFR *(rs1801131)*, ADRB3 *(rs4994) (Table 2). Plasma fibrinogen levels were significantly associated with *TNF *(rs1800750), though not after adjustment for multiple testing (Table 3). Serum uric acid levels were significantly associated with *CRP *(rs1417938) and *TNF *(rs361525), though also not after correction for multiple testing (Table 4). Serum homocysteine levels were significantly associated with *F2 *(rs1799963)*, MTHFR *(rs1801131, rs1801133, rs2066470) and *ADRB2 *(rs1042713) (Table 5). However, only rs1801133 remained significant with an FDR-adjusted P value of 0.005. Compared with minimally adjusted models, most associations became more significant in fully adjusted models. The following data for the concentrations of the four biomarkers in relation to the 27 candidate SNPs from minimally-adjusted and fully adjusted models are shown in additional file 1 available online (URL): the adjusted least-square means (LSMEANS) and standard errors (SE), exponentiated adjusted LSMEANS (CI), and P values for Satterthwaite adjusted F-statistic.

**Table 2:** Sample size and adjusted geometric means (95% confidence intervals) of serum C-reactive protein (mg/dL)*

Genotype	Minimally adjusted model LSmean (95% CI)	P_unadjusted_	P_FDR-adjusted_	Fully adjusted model LSmean (95% CI)	P_unadjusted_	P_FDR-adjusted_
rs3093058 (*CRP*)		0.0013	0.018		0.0012	0.023
TT	0.45 (0.29,0.71)			0.44 (0.29,0.66)		
TA	0.42 (0.37,0.49)			0.41 (0.36,0.46)		
AA	0.32 (0.30,0.34)			0.32 (0.31,0.33)		
rs1205(*CRP*)		0.0012	0.018		0.0059	0.038
AA	0.28 (0.26,0.31)			0.29 (0.27, 0.31)		
AG	0.32 (0.30,0.34)			0.32 (0.31, 0.34)		
GG	0.34 (0.32,0.37)			0.34 (0.32, 0.35)		
rs1801131 (*MTHFR*)		0.35	0.71		0.0024	0.023
TT	0.33 (0.29,0.36)			0.30 (0.28,0.32)		
TC	0.32 (0.30,0.34)			0.32 (0.30,0.33)		
CC	0.33 (0.31,0.35)			0.33 (0.32,0.35)		
rs4994 (*ADRB3*)		0.022	0.19		0.0026	0.023
CC	0.29 (0.20,0.42)			0.29 (0.23, 0.38)		
CT	0.29 (0.27,0.31)			0.29 (0.28, 0.31)		
TT	0.33 (0.31,0.35)			0.33 (0.31, 0.34)		

Note. *Only associations with unadjusted P (i.e., not adjusted for FDR) ≤ 0.05 in fully adjusted models are presented. FDR = false discovery rate.

**Table 3:** Adjusted geometric means (95% confidence intervals) of plasma fibrinogen (g/L) *

Genotype	Minimally adjusted model LSmean (95% CI)	P_unadjusted_	P_FDR-adjusted_	Fully adjusted model LSmean (95% CI)	P_unadjusted_	P_FDR-adjusted_
rs1800750 (*TNF*)		0.13	0.86		0.013	0.35
AA	3.2 (2.5,4.1)			2.8 (2.3,3.5)		
AG	3.4 (2.9,3.9)			3.5 (3.1,3.9)		
GG	3.0 (2.9,3.1)			3.0 (2.9,3.1)		

Note. *Only associations with unadjusted P (i.e., not adjusted for FDR) ≤ 0.05 in fully adjusted models are presented. FDR = false discovery rate.

**Table 4:** Adjusted geometric means (95% confidence intervals) of serum uric acid (umol/L)*

Genotype	Minimally adjusted model LSmean (95% CI)	P_unadjusted_	P_FDR-adjusted_	Fully adjusted model LSmean (95% CI)	P_unadjusted_	P_FDR-adjusted_
rs361525 (*TNF*)		0.05	0.76		0.04	0.55
AA	225 (185-275)			233 (195-279)		
AG	258 (248-268)			257 (246-268)		
GG	272 (267-278)			272 (268-276)		
rs1417938 (*CRP*)		0.16	0.76		0.03	0.55
TT	261 (248-273)			259 (250-270)		
TA	270 (265-274)			268 (263-273)		
AA	273 (267-278)			274 (268-279)		

Note. *Only associations with unadjusted P (i.e., not adjusted for FDR) ≤ 0.05 in fully adjusted models are presented. FDR = false discovery rate.

**Table 5:** Adjusted geometric means (95% confidence intervals) of serum homocysteine (umol/L)*

Genotype	Minimally adjusted model LSmean (95% CI)	P_unadjusted_	P_FDR-adjusted_	Fully adjusted model LSmean (95% CI)	P_unadjusted_	P_FDR-adjusted_
rs1801133 (*MTHFR*)		0.0002	0.0054		0.0002	0.0052
TT	9.9 (8.9-11.1)			9.8 (8.9-10.8)		
TC	8.2 (8.0-8.5)			8.2 (7.9-8.5)		
CC	7.7 (7.5-8.0)			7.7 (7.6-7.9)		
rs2066470 (*MTHFR*)		0.05	0.46		0.022	0.28
TT	8.2(7.4-9.1)			8.1 (7.1-9.3)		
TC	7.8 (7.5-8.1)			7.7 (7.5-8.0)		
CC	8.2 (8.0-8.4)			8.2 (8.0-8.4)		
rs1801131 (*MTHFR*)		0.02	0.28		0.05	0.28
CC	7.9 (7.4-8.4)			8.0 (7.3-8.8)		
CA	7.8 (7.6-8.1)			7.8 (7.5-8.1)		
AA	8.5 (8.1-8.8)			8.4 (8.1-8.7)		
rs1799963 (*F2*)		0.16	0.85		0.039	0.28
AA	-^a^	-		-		
AG	7.7 (7.1-8.4)			7.5 (6.9-8.2)		
GG	8.2 (8.0-8.4)			8.2 (7.9-8.3)		
rs1042713 (*ADRB2*)		0.17	0.85		0.04	0.28
AA	8.1 (7.7-8.6)			8.1 (7.7-8.5)		
AG	8.4 (8.1-8.7)			8.4 (8.1-8.7)		
GG	7.9 (7.6-8.2)			7.8 (7.5-8.2)		

Note. *Only associations with unadjusted P (i.e., not adjusted for FDR) ≤ 0.05 in fully adjusted models are presented. FDR = false discovery rate. ^a ^The frequency is zero for this genotype.

## Discussion

Cardiovascular diseases are multi-factorial as their pathogenesis is determined by genetic and environmental factors, as well as gene-gene and gene-environment interactions. This population-based genetic association study provides evidence that some intermediate CVD risk markers may be influenced by common genetic variants.

Numerous candidate gene studies have examined the role of inflammatory gene polymorphisms and the risk of CVD [41-45]. However, the findings remain inconsistent and the magnitude of associations remains modest [46]. C-Reactive protein is a systemic marker of inflammation and plays an important role in the pathogenesis of atherogenesis and its thrombotic complications. Plasma C-Reactive protein concentrations have been associated with *CRP *polymorphisms [42,43]. Although C-Reactive protein concentrations are a strong independent predictor of future vascular events, there has been no direct evidence that *CRP *variants contribute to cardiovascular disease phenotypes such as carotid intima-media thickness or arterial thrombosis [47-49].

Fibrinogen plays a key role in the final step of the coagulation cascade, i.e., the formation of fibrin; and it is a major determinant of plasma viscosity and erythrocyte aggregation. There is a large variation on estimates of the genetic heritability of plasma fibrinogen [44,45]. The researchers who estimated low heritability argued that environment, rather than genetic influences, has a greater effect on the level of plasma fibrinogen. It is also under debate whether plasma fibrinogen is a primary risk factor/mediator for coronary heart disease, or whether it is a marker for disease [50]. A large cohort study showed that fibrinogen may partly mediate the effects of other risk factors on carotid atherosclerosis, though it may not play a causal role [51]. The evidence from molecular biology seems to support the view that fibrinogen is a marker, rather than a mediator, of vascular disease [52]. Whether the association of plasma fibrinogen with the gene polymorphisms found in this report could be replicated in other genetic association studies remains unknown.

The findings that serum uric acid levels were associated with *CRP *and *TNF *polymorphisms need to be confirmed by other studies especially because the association was no longer significant after FDR adjustment. The underlying mechanisms need to be examined. In the literature, uric acid levels have been shown to be correlated with plasma levels of circulating TNF-alpha [53] and increased CRP expression [24]. Other genetic variants have been found to explain the variance in serum uric acid concentrations [54-56].

Plasma homocysteine is a thiol compound derived from methionine that is involved in two main metabolic pathways: the cycle of activated methyl groups, which requires folate and vitamin B12 as cofactors; and the transsulfuration pathway to cystathionine and cysteine, which requires vitamin B6 as a cofactor. Elevations in plasma homocysteine may be caused by genetic defects in enzymes involved in its metabolism or by deficiencies in cofactor levels [57]. Although the genetic influence of *MTHFR *polymorphisms on homocysteine levels is well-known, it is under debate whether the *MTHFR *polymorphism per se might be an independent contributor to cardiovascular risk [58].

There are some limitations in this study. First, the NHANES DNA bank was set up mainly to assess the allele frequency of these genes in a population-based sample, but it may not necessarily be one of the strong study designs to do genetic association studies. Second, our candidate genes were not selected based solely on explicit molecular/cellular biological pathways. For example, our study shows significant associations between *ADRB3 *and *MTHFR *genes to be associated with concentrations of serum C-reactive proteins although *ADRB3 *was mainly proposed to be a candidate gene for blood pressure and *MTHFR *was for serum homocysteine. The results are not surprising because of complex pathogenetic connections between immuno-inflammatory reactions, elevated homocysteine levels, and high blood pressure [59,60]. Third, the four biomarkers investigated in the study are largely influenced by environmental factors which may not be adequately captured by current study.

We did not investigate whether genetic and environmental factors modify each other in these associations. For example, hormone replacement therapy (especially estrogen) might be associated with increased inflammatory activity [61]. How genetic factors interact with inflammation-modulating effects of estrogen in causing adverse effects on atherogenesis or determining unfavorable clinical outcome is worthy of further investigation. Further studies are also needed to validate findings from recent genome-wide association studies that have revealed potential new SNPs [49,62,63].

## Conclusion

Our study provides some evidence that genetic factors contribute to the pathogenesis of inflammation and other CVD risk markers among adult women. Such knowledge may lead to improved prevention and treatment efforts. Identifying the variants that may modify the levels of these risk markers may allow for improved targeting and treatment of individuals or populations at an increased risk for future CVD events.

## Competing interests

The authors declare that they have no competing interests.

## Authors' contributions

AZF conceived of the study, and participated in its design and drafted the manuscript, AY performed the statistical analysis, MH helped drafting the manuscript, MC participated in the design of the study and guided in statistical methodology, JF participated in the design of the study, RN participated study design and interpretation of data, DH participated study design and interpretation of the data. NFD and AHM provided important comments to enrich the discussion. All authors read and approved the final manuscript.

## Pre-publication history

The pre-publication history for this paper can be accessed here:

http://www.biomedcentral.com/1471-2350/11/6/prepub

## Supplementary Material

Additional file 1 **Supplemental Tables**. Table S1. Exponentiated adjusted least-square means of concentrations of the four biomarkers (95% CIs) in relation to the 27 candidate SNPs from minimally-adjusted models. the adjusted least-square means (LSMEANS) and standard errors (SE), exponentiated adjusted LSMEANS (CI), and P values for Satterthwaite adjusted F-statistic are shown. Table S2. Exponentiated adjusted least-square means of concentrations of the four biomarkers (95% CIs) in relation to the 27 candidate SNPs from fully-adjusted models. the adjusted least-square means (LSMEANS) and standard errors (SE), exponentiated adjusted LSMEANS (CI), and P values for Satterthwaite adjusted F-statistic are shown. Click here for file
