# Supplementary material for: Gene polymorphisms in association with emerging cardiovascular risk markers in adult women
Source: BMC Med Genet. 2010 Jan 15;11:6. doi: 10.1186/1471-2350-11-6 (PMC2826307; doi:10.1186/1471-2350-11-6)
Supplement: Additional file 1 — Supplemental Tables. Table S1. Exponentiated adjusted least-square means of concentrations of the four biomarkers (95% CIs) in relation to the 27 candidate SNPs from minimally-adjusted models. the adjusted least-square means (LSMEANS) and standard errors (SE), exponentiated adjusted LSMEANS (CI), and P values for Satterthwaite adjusted F-statistic are shown. Table S2. Exponentiated adjusted least-square means of concentrations of the four biomarkers (95% CIs) in relation to the 27 candidate SNPs from fully-adjusted models. the adjusted least-square means (LSMEANS) and standard errors (SE), exponentiated adjusted LSMEANS (CI), and P values for Satterthwaite adjusted F-statistic are shown. [file 1471-2350-11-6-S1.DOC]

Table S1. Exponentiated adjusted least-square means of concentrations of the four biomarkers (95% CIs) in relation to the 27 candidate SNPs from minimally-adjusted models*

|  |  | C-reactive Protein (mg/dL) | | Plasma Fibrinogen (g/L) | | Serum Homocysteine (umol/L) | | Serum Uric Acid (umol/L) | |
| --- | --- | --- | --- | --- | --- | --- | --- | --- | --- |
| VARIANT | Genotype | Adjusted mean | P value |  | P value |  | P value |  | P value |
| *RS1042713 (ADRB2-01)* | AA | 0.33 (0.30,0.37) | 0.67 | 3.10 (3.01,3.20) | 0.24 | 8.13 (7.69,8.59) | 0.17 | 269.19 (257.57,281.34) | 0.42 |
|  | AG | 0.32 (0.30,0.34) |  | 2.97 (2.88,3.06) |  | 8.36 (8.06,8.67) |  | 272.86 (268.01,277.80) |  |
|  | GG | 0.33 (0.31,0.35) |  | 3.02 (2.90,3.14) |  | 7.92 (7.62,8.24) |  | 267.39 (261.81,273.09) |  |
| *RS1042714 (ADRB2-02)* | GG | 0.32 (0.30,0.35) | 0.42 | 3.03 (2.85,3.22) | 0.72 | 8.01 (7.41,8.65) | 0.69 | 265.98 (256.37,275.94) | 0.65 |
|  | GC | 0.32 (0.30,0.34) |  | 3.02 (2.92,3.13) |  | 8.21 (7.93,8.50) |  | 271.06 (264.70,277.58) |  |
|  | CC | 0.33 (0.31,0.36) |  | 2.98 (2.92,3.04) |  | 8.23 (8.01,8.45) |  | 271.52 (263.81,279.47) |  |
| *RS4994 (ADRB3-01)* | CC | 0.29 (0.20,0.42) | 0.02 | 3.22 (2.79,3.72) | 0.52 | 8.41 (7.51,9.42) | 0.40 | 254.17 (227.61,283.82) | 0.56 |
|  | CT | 0.29 (0.27,0.31) |  | 3.03 (2.94,3.13) |  | 7.97 (7.61,8.34) |  | 267.15 (256.19,278.59) |  |
|  | TT | 0.33 (0.31,0.35) |  | 3.01 (2.94,3.08) |  | 8.18 (7.99,8.38) |  | 270.84 (265.67,276.11) |  |
| *RS769214 (CAT-02)* | GG | 0.34 (0.28,0.40) | 0.79 | 3.09 (2.94,3.24) | 0.35 | 7.90 (7.41,8.43) | 0.22 | 272.86 (262.21,283.93) | 0.50 |
|  | GA | 0.32 (0.30,0.34) |  | 3.02 (2.93,3.10) |  | 8.06 (7.82,8.30) |  | 267.84 (262.75,273.02) |  |
|  | AA | 0.32 (0.30,0.35) |  | 2.98 (2.90,3.06) |  | 8.36 (8.04,8.69) |  | 271.82 (265.72,278.07) |  |
| *RS1205 (CRP-A)* | AA | 0.28 (0.26,0.31) | 0.0012 | 3.01 (2.89,3.14) | 0.96 | 8.23 (7.66,8.85) | 0.89 | 273.32 (259.89,287.46) | 0.86 |
|  | AG | 0.32 (0.30,0.34) |  | 3.00 (2.91,3.09) |  | 8.12 (7.84,8.40) |  | 269.79 (263.82,275.90) |  |
|  | GG | 0.34 (0.32,0.37) |  | 3.01 (2.94,3.08) |  | 8.19 (7.91,8.48) |  | 271.20 (264.72,277.83) |  |
| *RS1417938 (CRP-B)* | TT | 0.33 (0.29,0.39) | 0.35 | 2.98 (2.82,3.15) | 0.96 | 8.22 (7.74,8.73) | 0.91 | 260.71 (248.46,273.58) | 0.16 |
|  | TA | 0.34 (0.31,0.37) |  | 3.00 (2.92,3.09) |  | 8.17 (7.82,8.53) |  | 269.51 (264.91,274.19) |  |
|  | AA | 0.31 (0.30,0.33) |  | 3.01 (2.91,3.11) |  | 8.12 (7.90,8.35) |  | 272.53 (267.26,277.91) |  |
| *RS2808630 (CRP-C)* | GG | 0.32 (0.28,0.36) | 0.39 | 3.23 (3.02,3.45) | 0.042 | 8.10 (7.07,9.27) | 0.86 | 276.03 (258.29,294.99) | 0.58 |
|  | GA | 0.33 (0.30,0.36) |  | 2.96 (2.87,3.06) |  | 8.21 (7.84,8.60) |  | 270.93 (264.60,277.40) |  |
|  | AA | 0.31 (0.30,0.33) |  | 3.01 (2.93,3.08) |  | 8.29 (8.11,8.48) |  | 268.66 (263.67,273.74) |  |
| *RS1800947 (CRP-D)* † | *CC/CG* | 0.29 (0.25,0.32) | 0.056 | 3.09 (2.91,3.28) | 0.28 | 7.80 (6.95,8.76) | 0.40 | 268.71 (257.20,280.73) | 0.67 |
|  | *GG* | 0.33 (0.31,0.35) |  | 2.99 (2.92,3.06) |  | 8.21 (8.02,8.40) |  | 271.40 (266.72,276.16) |  |
| *RS3091244 (CRP-E)* | AA | 0.33 (0.27,0.41) | 0.09 | 3.18 (2.92,3.47) | 0.82 | 7.68 (6.75,8.75) | 0.78 | 262.11 (244.35,281.17) | 0.56 |
|  | AC | 0.32 (0.28,0.36) |  | 2.98 (2.82,3.15) |  | 8.27 (7.73,8.85) |  | 269.89 (259.82,280.34) |  |
|  | AT | 0.39 (0.32,0.48) |  | 3.03 (2.79,3.29) |  | 8.39 (7.49,9.40) |  | 274.76 (258.69,291.83) |  |
|  | CT | 0.34 (0.31,0.37) |  | 3.00 (2.92,3.09) |  | 8.14 (7.74,8.56) |  | 270.16 (265.76,274.63) |  |
|  | TT | 0.34 (0.29,0.40) |  | 2.98 (2.83,3.14) |  | 8.40 (7.84,9.00) |  | 262.39 (251.18,274.09) |  |
|  | CC | 0.30 (0.29,0.32) |  | 3.04 (2.96,3.13) |  | 8.06 (7.74,8.40) |  | 271.58 (264.82,278.51) |  |
| *RS3093058 (CRP-F)* | TT | 0.45 (0.29,0.71) | 0.0013 | 3.08 (2.61,3.62) | 0.70 | 8.38 (6.62,10.61) | 0.59 | 271.33 (248.96,295.71) | 0.37 |
|  | TA | 0.42 (0.37,0.49) |  | 3.07 (2.88,3.27) |  | 7.91 (7.56,8.28) |  | 275.16 (265.73,284.92) |  |
|  | AA | 0.32 (0.30,0.34) |  | 3.01 (2.94,3.07) |  | 8.13 (7.97,8.30) |  | 269.70 (265.68,273.77) |  |
| *RS3093066 (CRP-G)* | AA | 0.34 (0.27,0.44) | 0.93 | 3.39 (3.11,3.69) | 0.22 | 7.94 (6.57,9.58) | 0.94 | 280.94 (262.06,301.17) | 0.56 |
|  | AC | 0.33 (0.29,0.36) |  | 2.98 (2.79,3.18) |  | 8.18 (7.55,8.85) |  | 273.29 (262.98,284.01) |  |
|  | CC | 0.33 (0.31,0.35) |  | 3.02 (2.95,3.09) |  | 8.13 (7.97,8.30) |  | 269.73 (265.36,274.16) |  |
| *RS1799963 (F2-01)* | AG | 0.37 (0.30,0.45) | 0.22 | 3.00 (2.87,3.14) | 0.91 | 7.69 (7.05,8.38) | 0.16 | 275.48 (251.74,301.46) | 0.68 |
|  | GG | 0.32 (0.31,0.34) |  | 3.01 (2.94,3.08) |  | 8.18 (8.01,8.36) |  | 270.22 (266.08,274.42) |  |
| *RS6025 (F5-01)* † | *AA/AG* | 0.34 (0.30,0.38) | 0.49 | 3.02 (2.87,3.18) | 0.95 | 7.88 (7.37,8.42) | 0.27 | 274.37 (253.78,296.63) | 0.68 |
|  | *GG* | 0.32 (0.31,0.34) |  | 3.01 (2.94,3.09) |  | 8.17 (8.01,8.33) |  | 270.11 (266.32,273.95) |  |
| *RS1800790 (FGB-01)* | AA | 0.32 (0.25,0.41) | 0.97 | 3.15 (2.81,3.54) | 0.21 | 7.83 (6.69,9.18) | 0.49 | 256.66 (233.50,282.13) | 0.51 |
|  | AG | 0.32 (0.29,0.35) |  | 3.08 (2.96,3.21) |  | 7.99 (7.60,8.41) |  | 270.35 (261.83,279.15) |  |
|  | GG | 0.32 (0.31,0.34) |  | 2.98 (2.91,3.05) |  | 8.24 (8.06,8.41) |  | 270.79 (266.51,275.14) |  |
| *RS5918 (ITGB3-01)* | CC | 0.36 (0.23,0.56) | 0.63 | 2.97 (2.56,3.44) | 0.90 | 8.77 (7.28,10.55) | 0.59 | 288.52 (268.46,310.07) | 0.10 |
|  | CT | 0.33 (0.31,0.35) |  | 2.99 (2.88,3.11) |  | 8.09 (7.81,8.37) |  | 275.48 (268.72,282.41) |  |
|  | TT | 0.32 (0.30,0.34) |  | 3.02 (2.94,3.09) |  | 8.18 (7.97,8.39) |  | 268.03 (262.31,273.88) |  |
| *RS1801131 (MTHFR-01)* | CC | 0.32 (0.27,0.37) | 0.81 | 2.99 (2.75,3.24) | 0.87 | 7.88 (7.38,8.42) | 0.021 | 267.79 (259.37,276.49) | 0.85 |
|  | CA | 0.33 (0.30,0.35) |  | 3.02 (2.94,3.10) |  | 7.83 (7.58,8.08) |  | 270.46 (263.36,277.75) |  |
|  | AA | 0.32 (0.31,0.34) |  | 3.01 (2.94,3.09) |  | 8.46 (8.12,8.82) |  | 270.50 (265.76,275.33) |  |
| *RS1801133 (MTHFR-02)* | TT | 0.33 (0.29,0.36) | 0.35 | 3.08 (2.93,3.22) | 0.54 | 9.93 (8.90,11.08) | 0.0002 | 275.99 (265.40,287.00) | 0.61 |
|  | TC | 0.32 (0.30,0.34) |  | 3.02 (2.95,3.09) |  | 8.24 (7.97,8.53) |  | 269.61 (264.33,275.00) |  |
|  | CC | 0.33 (0.31,0.35) |  | 2.99 (2.89,3.09) |  | 7.74 (7.53,7.95) |  | 269.29 (261.75,277.05) |  |
| *RS2066470 (MTHFR-03)* | TT | 0.32 (0.22,0.47) | 0.67 | 2.96 (2.71,3.24) | 0.92 | 8.20 (7.40,9.09) | 0.051 | 282.54 (252.68,315.94) | 0.65 |
|  | TC | 0.33 (0.31,0.36) |  | 3.02 (2.87,3.17) |  | 7.80 (7.51,8.11) |  | 267.76 (257.12,278.83) |  |
|  | CC | 0.32 (0.30,0.34) |  | 3.01 (2.96,3.07) |  | 8.23 (8.02,8.44) |  | 270.48 (266.12,274.91) |  |
| *RS1799983 (NOS3-01)* | TT | 0.30 (0.27,0.33) | 0.27 | 3.10 (2.88,3.33) | 0.13 | 8.75 (7.80,9.82) | 0.21 | 270.34 (256.51,284.91) | 0.46 |
|  | TG | 0.32 (0.30,0.35) |  | 3.07 (3.00,3.15) |  | 8.25 (8.00,8.52) |  | 272.95 (265.91,280.19) |  |
|  | GG | 0.33 (0.31,0.35) |  | 2.95 (2.85,3.05) |  | 8.00 (7.74,8.26) |  | 267.86 (263.69,272.09) |  |
| *RS2070744 (NOS3-11)* | CC | 0.32 (0.30,0.35) | 0.98 | 3.07 (2.91,3.24) | 0.35 | 8.10 (7.39,8.88) | 0.97 | 271.98 (261.09,283.32) | 0.64 |
|  | CT | 0.33 (0.31,0.34) |  | 3.04 (2.94,3.14) |  | 8.17 (7.92,8.43) |  | 271.87 (265.14,278.76) |  |
|  | TT | 0.32 (0.30,0.35) |  | 2.96 (2.88,3.05) |  | 8.16 (7.89,8.44) |  | 267.92 (262.28,273.67) |  |
| *RS662 (PON1-01)* | GG | 0.30 (0.27,0.33) | 0.13 | 2.98 (2.84,3.13) | 0.81 | 8.03 (7.68,8.38) | 0.65 | 263.37 (250.68,276.69) | 0.34 |
|  | GA | 0.33 (0.30,0.36) |  | 3.01 (2.92,3.09) |  | 8.14 (7.90,8.38) |  | 270.97 (265.26,276.81) |  |
|  | AA | 0.33 (0.31,0.34) |  | 3.03 (2.92,3.15) |  | 8.24 (7.95,8.53) |  | 272.32 (267.25,277.49) |  |
| *RS854560 (PON1-02)* | AA | 0.30 (0.27,0.33) | 0.23 | 2.93 (2.82,3.04) | 0.40 | 8.36 (7.72,9.05) | 0.65 | 268.43 (257.32,280.02) | 0.49 |
|  | AT | 0.33 (0.31,0.35) |  | 3.03 (2.93,3.12) |  | 8.18 (7.94,8.43) |  | 273.29 (268.25,278.42) |  |
|  | TT | 0.33 (0.31,0.35) |  | 3.01 (2.94,3.09) |  | 8.09 (7.87,8.31) |  | 268.44 (261.68,275.36) |  |
| *RS1801282 (PPARG-11)* | GG | 0.29 (0.23,0.37) | 0.39 | 2.88 (2.55,3.26) | 0.46 | 8.30 (6.85,10.05) | 0.97 | 268.07 (246.14,291.95) | 0.48 |
|  | GC | 0.31 (0.28,0.35) |  | 3.06 (2.95,3.17) |  | 8.14 (7.74,8.56) |  | 266.46 (257.78,275.44) |  |
|  | CC | 0.33 (0.31,0.35) |  | 3.01 (2.94,3.08) |  | 8.17 (7.97,8.38) |  | 271.48 (266.87,276.17) |  |
| *RS4986790 (TLR4-01)* | GG | 0.32 (0.23,0.45) | 0.20 | 2.71 (2.42,3.03) | 0.47 | 9.40 (8.75,10.09) | 0.33 | 322.35 (271.76,382.36) | 0.33 |
|  | GA | 0.36 (0.31,0.42) |  | 3.02 (2.87,3.17) |  | 8.13 (7.65,8.63) |  | 268.47 (254.47,283.24) |  |
|  | AA | 0.32 (0.30,0.34) |  | 3.01 (2.95,3.07) |  | 8.14 (7.95,8.33) |  | 270.02 (264.32,275.84) |  |
| *RS1800750 (TNF-01)* | AA | 0.66 (0.12,3.50) | 0.61 | 3.20 (2.52,4.06) | 0.13 | 7.75 (7.28,8.25) | 0.92 | 287.59 (232.24,356.13) | 0.83 |
|  | AG | 0.34 (0.26,0.44) |  | 3.37 (2.92,3.88) |  | 8.18 (6.84,9.78) |  | 266.87 (242.17,294.08) |  |
|  | GG | 0.32 (0.31,0.34) |  | 3.00 (2.94,3.07) |  | 8.15 (7.98,8.31) |  | 270.16 (265.69,274.71) |  |
| *RS1800629 (TNF-02)* | AA | 0.31 (0.25,0.38) | 0.73 | 2.95 (2.74,3.18) | 0.33 | 8.59 (7.25,10.16) | 0.34 | 274.77 (252.62,298.86) | 0.47 |
|  | AG | 0.33 (0.30,0.36) |  | 2.97 (2.88,3.06) |  | 7.92 (7.67,8.17) |  | 266.95 (260.94,273.09) |  |
|  | GG | 0.32 (0.31,0.34) |  | 3.04 (2.96,3.11) |  | 8.22 (7.96,8.48) |  | 271.54 (266.63,276.53) |  |
| *RS361525 (TNF-04)* | AA | 0.26 (0.20,0.35) | 0.35 | 3.00 (2.57,3.50) | 0.31 | 7.27 (5.26,10.03) | 0.47 | 225.46 (184.86,274.99) | 0.050 |
|  | AG | 0.33 (0.30,0.37) |  | 3.14 (2.90,3.41) |  | 7.88 (7.18,8.64) |  | 257.55 (247.83,267.66) |  |
|  | GG | 0.32 (0.31,0.34) |  | 3.00 (2.93,3.07) |  | 8.20 (8.02,8.37) |  | 272.25 (266.90,277.71) |  |

Note. *Estimates were obtained from multiple linear regression models. The models were adjusted for race/ethnicity only. P values were from the Satterthwaite adjusted F-statistic. † Due to very low frequencies of the minor allele, a dominant model of inheritance was assumed to generate estimates. CI=confidence interval. SNP=Single-nucleotide polymorphism.

Table S2. Exponentiated adjusted least-square means of concentrations of the four biomarkers (95% CIs) in relation to the 27 candidate SNPs from fully-adjusted models*

|  |  | C-reactive Protein (mg/dL) | | Plasma Fibrinogen (g/L) | | Serum Homocysteine (umol/L) | | Serum Uric Acid (umol/L) | |
| --- | --- | --- | --- | --- | --- | --- | --- | --- | --- |
| VARIANT | Genotype |  | P value |  | P value |  | P value |  | P value |
| *RS1042713 (ADRB2-01)* | AA | 0.34 (0.31,0.37) | 0.24 | 3.14 (3.04,3.23) | 0.098 | 8.06 (7.65,8.49) | 0.045 | 271.83 (263.67,280.24) | 0.11 |
|  | AG | 0.32 (0.30,0.33) |  | 2.97 (2.88,3.06) |  | 8.40 (8.10,8.70) |  | 272.94 (268.29,277.68) |  |
|  | GG | 0.32 (0.30,0.34) |  | 3.00 (2.90,3.12) |  | 7.84 (7.54,8.17) |  | 265.63 (259.95,271.43) |  |
| *RS1042714 (ADRB2-02)* | GG | 0.32 (0.30,0.34) | 0.07 | 3.02 (2.85,3.20) | 0.79 | 7.97 (7.51,8.46) | 0.59 | 263.82 (254.55,273.43) | 0.33 |
|  | GC | 0.31 (0.30,0.33) |  | 3.02 (2.93,3.11) |  | 8.20 (7.90,8.50) |  | 271.14 (265.06,277.35) |  |
|  | CC | 0.34 (0.32,0.36) |  | 2.98 (2.93,3.04) |  | 8.19 (7.98,8.40) |  | 271.92 (266.32,277.64) |  |
| *RS4994 (ADRB3-01)* | CC | 0.29 (0.23,0.38) | 0.003 | 3.22 (2.87,3.61) | 0.36 | 8.28 (6.99,9.81) | 0.59 | 255.45 (212.06,307.73) | 0.75 |
|  | CT | 0.29 (0.28,0.31) |  | 3.04 (2.97,3.11) |  | 7.98 (7.60,8.37) |  | 267.83 (257.83,278.21) |  |
|  | TT | 0.33 (0.31,0.34) |  | 3.00 (2.94,3.07) |  | 8.14 (7.93,8.36) |  | 270.49 (265.97,275.09) |  |
| *RS769214 (CAT-02)* | GG | 0.33 (0.28,0.39) | 0.72 | 3.08 (2.96,3.20) | 0.11 | 7.92 (7.37,8.52) | 0.50 | 271.45 (265.06,277.98) | 0.91 |
|  | GA | 0.32 (0.31,0.34) |  | 3.03 (2.95,3.12) |  | 8.07 (7.81,8.34) |  | 269.87 (264.36,275.49) |  |
|  | AA | 0.32 (0.30,0.34) |  | 2.96 (2.91,3.02) |  | 8.26 (7.95,8.58) |  | 269.48 (263.97,275.11) |  |
| *RS1205 (CRP-A)* | AA | 0.29 (0.27,0.31) | 0.006 | 2.97 (2.84,3.11) | 0.89 | 8.38 (7.82,8.99) | 0.61 | 277.53 (263.59,292.21) | 0.43 |
|  | AG | 0.32 (0.30,0.34) |  | 3.01 (2.92,3.10) |  | 8.11 (7.84,8.39) |  | 270.33 (265.30,275.45) |  |
|  | GG | 0.34 (0.32,0.35) |  | 3.00 (2.94,3.06) |  | 8.11 (7.84,8.39) |  | 268.82 (263.23,274.54) |  |
| *RS1417938 (CRP-B)* | TT | 0.33 (0.28,0.39) | 0.33 | 3.01 (2.84,3.18) | 0.99 | 7.95 (7.44,8.50) | 0.77 | 259.39 (249.53,269.64) | 0.030 |
|  | TA | 0.33 (0.31,0.36) |  | 3.00 (2.92,3.07) |  | 8.12 (7.77,8.48) |  | 267.77 (263.01,272.61) |  |
|  | AA | 0.31 (0.30,0.33) |  | 3.00 (2.91,3.10) |  | 8.15 (7.94,8.37) |  | 273.71 (268.29,279.24) |  |
| *RS2808630 (CRP-C)* | GG | 0.31 (0.27,0.36) | 0.88 | 3.23 (3.00,3.47) | 0.059 | 8.00 (7.19,8.91) | 0.49 | 273.83 (256.56,292.26) | 0.75 |
|  | GA | 0.32 (0.30,0.35) |  | 2.96 (2.86,3.06) |  | 8.13 (7.84,8.43) |  | 268.85 (263.46,274.35) |  |
|  | AA | 0.32 (0.31,0.33) |  | 3.01 (2.94,3.08) |  | 8.33 (8.14,8.52) |  | 270.21 (266.41,274.07) |  |
| *RS1800947 (CRP-D)†* | CC/CG | 0.30 (0.27,0.33) | 0.17 | 3.07 (2.90,3.25) | 0.36 | 7.86 (7.08,8.72) | 0.47 | 273.02 (261.90,284.61) | 0.72 |
|  | GG | 0.33 (0.31,0.34) |  | 2.99 (2.92,3.06) |  | 8.17 (7.95,8.41) |  | 270.82 (266.93,274.77) |  |
| *RS3091244 (CRP-E)* | AA | ‡ |  | 3.24 (3.03,3.46) | 0.90 | ‡ |  | 270.96 (246.72,297.58) | 0.30 |
|  | AC |  |  | 3.02 (2.85,3.20) |  |  |  | 272.99 (264.73,281.50) |  |
|  | AT |  |  | 3.00 (2.79,3.23) |  |  |  | 276.88 (264.04,290.33) |  |
|  | CT |  |  | 3.00 (2.92,3.07) |  |  |  | 268.01 (262.57,273.56) |  |
|  | TT |  |  | 3.01 (2.86,3.17) |  |  |  | 261.00 (252.31,269.98) |  |
|  | CC |  |  | 3.03 (2.94,3.12) |  |  |  | 272.02 (264.83,279.39) |  |
| *RS3093058 (CRP-F)* | TT | 0.44 (0.29,0.66) | 0.0012 | 3.15 (2.71,3.67) | 0.74 | 8.80 (7.12,10.87) | 0.46 | 267.71 (244.17,293.53) | 0.51 |
|  | TA | 0.41 (0.36,0.46) |  | 3.04 (2.87,3.23) |  | 7.94 (7.61,8.27) |  | 274.05 (265.11,283.29) |  |
|  | AA | 0.32 (0.31,0.33) |  | 3.01 (2.95,3.07) |  | 8.10 (7.92,8.29) |  | 269.46 (265.98,272.98) |  |
| *RS3093066 (CRP-G)* | AA | 0.37 (0.30,0.46) | 0.41 | 3.39 (3.11,3.69) | 0.20 | 8.24 (7.17,9.48) | 0.85 | 294.52 (274.17,316.38) | 0.11 |
|  | AC | 0.34 (0.30,0.38) |  | 3.04 (2.88,3.22) |  | 7.98 (7.40,8.62) |  | 271.23 (262.52,280.22) |  |
|  | CC | 0.33 (0.31,0.34) |  | 3.01 (2.95,3.08) |  | 8.11 (7.94,8.28) |  | 269.55 (265.28,273.88) |  |
| *RS1799963 (F2-01)* | AG | 0.37 (0.32,0.42) | 0.10 | 3.03 (2.88,3.18) | 0.80 | 7.48 (6.86,8.15) | 0.039 | 280.04 (261.80,299.56) | 0.30 |
|  | GG | 0.32 (0.31,0.34) |  | 3.01 (2.95,3.07) |  | 8.16 (7.98,8.34) |  | 269.92 (266.08,273.81) |  |
| *RS6025 (F5-01)†* | AA/AG | 0.33 (0.29,0.36) | 0.85 | 2.96 (2.84,3.09) | 0.49 | 8.09 (7.52,8.70) | 0.89 | 272.37 (252.51,293.80) | 0.81 |
|  | GG | 0.32 (0.31,0.34) |  | 3.02 (2.95,3.08) |  | 8.13 (7.95,8.32) |  | 270.01 (266.50,273.58) |  |
| *RS1800790 (FGB-01)* | AA | 0.28 (0.23,0.34) | 0.36 | 3.11 (2.76,3.51) | 0.23 | 7.66 (6.57,8.94) | 0.63 | 255.31 (227.38,286.68) | 0.52 |
|  | AG | 0.33 (0.30,0.36) |  | 3.08 (2.98,3.19) |  | 8.07 (7.68,8.47) |  | 271.54 (262.95,280.41) |  |
|  | GG | 0.32 (0.31,0.33) |  | 2.98 (2.92,3.04) |  | 8.17 (7.97,8.37) |  | 270.09 (265.83,274.42) |  |
| *RS5918 (ITGB3-01)* | CC | 0.35 (0.24,0.52) | 0.83 | 2.90 (2.56,3.28) | 0.70 | 8.70 (7.29,10.39) | 0.65 | 281.13 (265.57,297.59) | 0.29 |
|  | CT | 0.33 (0.31,0.35) |  | 2.99 (2.89,3.10) |  | 8.08 (7.78,8.38) |  | 273.22 (265.43,281.25) |  |
|  | TT | 0.32 (0.31,0.34) |  | 3.02 (2.96,3.08) |  | 8.14 (7.91,8.39) |  | 268.71 (264.35,273.15) |  |
| *RS1801131 (MTHFR-01)* | CC | 0.33 (0.29,0.38) | 0.60 | 3.00 (2.76,3.25) | 0.75 | 8.01 (7.29,8.80) | 0.054 | 270.22 (259.98,280.86) | 0.98 |
|  | CA | 0.33 (0.31,0.34) |  | 3.03 (2.96,3.10) |  | 7.79 (7.50,8.10) |  | 269.80 (264.30,275.42) |  |
|  | AA | 0.32 (0.31,0.33) |  | 3.00 (2.94,3.06) |  | 8.40 (8.11,8.69) |  | 270.23 (265.90,274.63) |  |
| *RS1801133 (MTHFR-02)* | TT | 0.30 (0.28,0.32) | 0.0024 | 3.03 (2.92,3.14) | 0.92 | 9.77 (8.86,10.76) | 0.0002 | 269.52 (260.18,279.20) | 0.99 |
|  | TC | 0.32 (0.30,0.33) |  | 3.01 (2.94,3.08) |  | 8.18 (7.89,8.49) |  | 269.85 (264.84,274.95) |  |
|  | CC | 0.33 (0.32,0.35) |  | 3.01 (2.93,3.09) |  | 7.74 (7.56,7.93) |  | 270.19 (264.84,275.64) |  |
| *RS2066470 (MTHFR-03)* | TT | 0.32 (0.24,0.42) | 0.80 | 2.75 (2.50,3.04) | 0.30 | 8.12 (7.12,9.26) | 0.022 | 269.21 (249.03,291.03) | 0.31 |
|  | TC | 0.33 (0.30,0.36) |  | 3.00 (2.87,3.13) |  | 7.74 (7.46,8.03) |  | 264.53 (254.61,274.83) |  |
|  | CC | 0.32 (0.31,0.34) |  | 3.02 (2.96,3.08) |  | 8.20 (7.99,8.42) |  | 271.04 (266.88,275.27) |  |
| *RS1799983 (NOS3-01)* | TT | 0.30 (0.27,0.32) | 0.22 | 3.10 (2.91,3.31) | 0.060 | 8.67 (7.72,9.75) | 0.27 | 271.58 (257.01,286.98) | 0.38 |
|  | TG | 0.32 (0.31,0.34) |  | 3.08 (3.01,3.15) |  | 8.20 (7.95,8.45) |  | 273.02 (267.44,278.71) |  |
|  | GG | 0.33 (0.31,0.35) |  | 2.95 (2.87,3.03) |  | 8.01 (7.76,8.26) |  | 267.58 (263.10,272.14) |  |
| *RS2070744 (NOS3-11)* | CC | 0.33 (0.31,0.36) | 0.78 | 3.09 (2.95,3.22) | 0.12 | 8.05 (7.33,8.85) | 0.85 | 275.70 (266.42,285.31) | 0.16 |
|  | CT | 0.32 (0.31,0.34) |  | 3.03 (2.96,3.12) |  | 8.17 (7.95,8.40) |  | 271.95 (265.92,278.11) |  |
|  | TT | 0.32 (0.30,0.34) |  | 2.95 (2.88,3.03) |  | 8.10 (7.85,8.36) |  | 266.22 (261.45,271.09) |  |
| *RS662 (PON1-01)* | GG | 0.30 (0.29,0.32) | 0.19 | 3.03 (2.92,3.14) | 0.65 | 8.16 (7.84,8.49) | 0.55 | 266.14 (257.16,275.44) | 0.58 |
|  | GA | 0.33 (0.31,0.35) |  | 2.99 (2.92,3.06) |  | 8.05 (7.79,8.31) |  | 270.97 (265.62,276.42) |  |
|  | AA | 0.33 (0.31,0.34) |  | 3.03 (2.92,3.15) |  | 8.21 (7.93,8.49) |  | 270.84 (265.24,276.55) |  |
| *RS854560 (PON1-02)* | AA | 0.30 (0.27,0.35) | 0.40 | 2.94 (2.82,3.07) | 0.50 | 8.32 (7.63,9.06) | 0.72 | 267.90 (256.15,280.19) | 0.71 |
|  | AT | 0.32 (0.31,0.34) |  | 3.02 (2.94,3.11) |  | 8.12 (7.91,8.33) |  | 271.79 (266.57,277.11) |  |
|  | TT | 0.33 (0.31,0.34) |  | 3.01 (2.94,3.08) |  | 8.09 (7.83,8.36) |  | 269.57 (264.55,274.68) |  |
| *RS1801282 (PPARG-11)* | GG | 0.31 (0.26,0.37) | 0.25 | 2.94 (2.59,3.34) | 0.46 | 8.17 (6.86,9.72) | 0.88 | 261.68 (233.04,293.85) | 0.39 |
|  | GC | 0.31 (0.28,0.34) |  | 3.06 (2.95,3.18) |  | 8.20 (7.81,8.62) |  | 266.02 (258.22,274.05) |  |
|  | CC | 0.33 (0.31,0.34) |  | 3.00 (2.94,3.06) |  | 8.12 (7.91,8.33) |  | 271.49 (267.23,275.81) |  |
| *RS4986790 (TLR4-01)* | GG | 0.30 (0.24,0.36) | 0.15 | 2.79 (2.53,3.08) | 0.53 | 9.00 (7.93,10.20) | 0.46 | 326.59 (290.52,367.13) | 0.18 |
|  | GA | 0.36 (0.31,0.41) |  | 3.02 (2.90,3.15) |  | 8.19 (7.76,8.64) |  | 271.51 (258.87,284.77) |  |
|  | AA | 0.32 (0.31,0.33) |  | 3.01 (2.96,3.06) |  | 8.10 (7.90,8.30) |  | 269.35 (264.22,274.57) |  |
| *RS1800750 (TNF-01)* | AA | 0.63 (0.14,2.94) | 0.52 | 2.80 (2.26,3.47) | 0.013 | 7.16 (5.85,8.77) | 0.78 | 272.61 (236.51,314.22) | 0.95 |
|  | AG | 0.32 (0.26,0.39) |  | 3.50 (3.12,3.93) |  | 8.12 (7.04,9.36) |  | 270.96 (249.58,294.18) |  |
|  | GG | 0.32 (0.31,0.34) |  | 3.00 (2.94,3.06) |  | 8.12 (7.93,8.31) |  | 269.86 (265.85,273.93) |  |
| *RS1800629 (TNF-02)* | AA | 0.30 (0.26,0.36) | 0.58 | 3.02 (2.85,3.21) | 0.38 | 8.65 (7.05,10.62) | 0.52 | 281.93 (260.81,304.77) | 0.45 |
|  | AG | 0.33 (0.31,0.35) |  | 2.97 (2.88,3.07) |  | 7.98 (7.79,8.18) |  | 268.42 (260.71,276.35) |  |
|  | GG | 0.32 (0.31,0.34) |  | 3.03 (2.97,3.10) |  | 8.14 (7.88,8.41) |  | 270.36 (266.50,274.28) |  |
| *RS361525 (TNF-04)* | AA | 0.29 (0.22,0.39) | 0.66 | 2.82 (2.49,3.18) | 0.08 | 8.21 (6.17,10.93) | 0.76 | 233.01 (194.94,278.52) | 0.041 |
|  | AG | 0.33 (0.30,0.37) |  | 3.20 (2.97,3.45) |  | 7.95 (7.38,8.56) |  | 256.86 (245.93,268.28) |  |
|  | GG | 0.32 (0.31,0.34) |  | 2.99 (2.94,3.05) |  | 8.15 (7.96,8.34) |  | 271.95 (267.80,276.15) |  |

Note. * The estimates were obtained from multiple linear regression models. Covariates/potential confounders included age, race/ethnicity, education, menopausal status, female hormone use, smoking status, alcohol consumption, dietary fiber intake, total energy intake, physical activity, body mass index, and aspirin use. However, only significant covariates were retained in fully-adjusted models for a specific inflammation marker predicted by certain genetic variants. P values were from the Satterthwaite adjusted F-statistic. † Due to very low frequencies of the minor allele, a dominant model of inheritance was assumed to generate estimates. ‡ estimates are not available due to low frequency of the minor allele and assuming different inheritance model is inappropriate for tri-allelic genes. CI=confidence interval. SNP=Single-nucleotide polymorphism.
